# DNMT1-PPARγ pathway in macrophages regulates chronic inflammation and atherosclerosis development in mice

**DOI:** 10.1038/srep30053

**Published:** 2016-08-17

**Authors:** Jie Yu, Youzhu Qiu, Jie Yang, Shizhu Bian, Guozhu Chen, Mengyang Deng, Huali Kang, Lan Huang

**Affiliations:** 1 Institute of Cardiovascular Diseases of the PLA , Xinqiao Hospital, Third Military Medical University, Chongqing, 400037, China

## Abstract

The DNA methyltransferase-mediated proinflammatory activation of macrophages is causally linked to the development of atherosclerosis (AS). However, the role of DNMT1, a DNA methylation maintenance enzyme, in macrophage polarization and AS development remains obscure. Here, we established transgenic mice with macrophage-specific overexpression of DNMT1 (Tg^DNMT1^) or PPAR-γ (Tg^PPAR-γ^) to investigate their effects on AS progression in ApoE-knockout mice fed an atherogenic diet. Primary macrophages were extracted to study the role of the DNMT1/PPAR-γ pathway in regulating inflammatory cytokine production. We demonstrated that Tg^DNMT1^ significantly increased proinflammatory cytokine production in macrophages and plasma, and it accelerated the progression of AS in the atherogenic diet-treated ApoE-knockout mice. Further, we found that the DNA methylation status of the proximal PPAR-γ promoter was regulated by DNMT1 in macrophages. Notably, additional Tg^PPAR-γ^ or pharmacological activation of PPAR-γ effectively prevented Tg^DNMT1^-induced proinflammatory cytokine production in macrophages and AS development in the mouse model. Finally, we demonstrated that elevated DNMT1 was correlated with decreased PPAR-γ, and increased proinflammatory cytokine production in the peripheral blood monocytes isolated from the patients with AS, compared to those of healthy donors. Our findings shed light on a novel strategy for the prevention and therapy of AS.

Atherosclerosis (AS), a common disorder worldwide, is causally linked to complex diseases, including coronary heart disease and stroke^1^,^2^,^3^. Macrophage-mediated inflammation responses play a pivotal role in the development of AS^4^,^5^. The accumulation of oxidative low-density lipoprotein (ox-LDL) and macrophages in the arterial wall plays an important role in the pathophysiology of AS development by determining plaque formation, progression and rupture^6^,^7^. In the early stages of AS, monocytes in the circulation are attracted to the endothelium of the blood vessels. Vascular cell adhesion molecules and monocyte chemoattractant protein are involved in the chemotaxis of monocytes^1^,^3^. The macrophages, which are differentiated from monocytes in the intima, promote the development of AS by secreting proinflammatory cytokines (e.g., TNFα, IL-6 and IL-1β), taking up cholesterol and initiating apoptosis^1^,^3^,^8^. Suppressing the proinflammatory activity of macrophages is an effective strategy for the prevention and cure of AS^3^.

Macrophages can be classically activated (M1) or alternatively activated (M2)^9^,^10^. The M1 macrophages are prone to a proinflammatory phenotype, expressing high levels of proinflammatory cytokines (e.g., TNFα, IL-6 and IL-1β), while the M2 macrophages play an anti-inflammatory role by expressing high levels of anti-inflammatory cytokines (e.g., IL-10 and Arg1)^10^. Previous studies have revealed that a high fat diet (HFD) or obesity could induce the M1 macrophages, while Th2 cytokines, such as IL-4 or IL-13, could polarize the M2 macrophages^9^. Peroxisome proliferator-activated receptor-gamma (PPAR-γ) also plays an important role in promoting the polarization of M2 macrophages^11^,^12^. Regulation of PPAR-γ expression or activity provides an effective means to manage inflammatory cytokine production by macrophages^11^,^12^.

Emerging studies have focused on the role of gene expression in the regulation of macrophage activity^8^,^13^,^14^. However, the epigenetic regulation of macrophage activity is still not fully understood. Epigenetic regulation, including DNA methylation, links environmental factors (e.g., stress, diet) to complex disorders (e.g., AS, cancer)^15^,^16^,^17^. DNA methylation of cytokines, primarily at CpG dinucleotide sites, is the most common epigenetic modification of the genome^18^,^19^. CpGs are often enriched in the promoter regions and in the first exon/5′-untranslated regions of genes^20^. Promoters of transcriptionally active genes are typically hypomethylated, whereas DNA hypermethylation can result in gene silencing^15^,^19^. De novo methylation is mediated by DNA methyltransferases (DNMTs) 3a and 3b^21^. Once established, DNA methylation is then maintained through mitosis, primarily by the maintenance enzyme DNMT1^22^,^23^. Altering global DNA methylation could regulate chronic inflammation-associated disease^16^,^17^. However, the roles of DNA methyltransferases in macrophage polarization and AS development have not been fully elucidated.

Here in this study, we hypothesized that DNMT1 could regulate inflammatory cytokine production in macrophages and affect the development of AS. To test this hypothesis, we established a mouse model with a macrophage-specific transgene of DNMT1 or PPAR-γ. We also established an AS mouse model by feeding ApoE-knockout (ApoE^−/−^) mice with an atherogenic diet. Using *in vitro* and *in vivo* experiments, we identified the role of the DNMT1/PPAR-γ pathway in the development of AS.

## Results

### Ectopic expression of DNMT1 in atherosclerosis-associated macrophages

**Figure 1:**
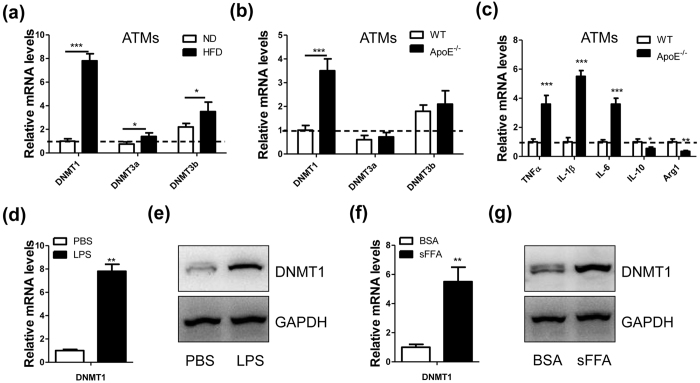
Elevated DNMT1 levels in atherosclerosis-associated macrophages. (**a**) mRNA levels of DNMT1, DNMT3a and DNMT3b in adipose tissue-derived macrophages (ATMs) from male C57BL/6 mice fed a normal diet (ND) or a high fat diet (HFD) for 12 weeks (n = 5, *P < 0.05 and ***P < 0.005). (**b**) mRNA levels of DNMT1, DNMT3a and DNMT3b in the ATMs of male wild-type (WT) or ApoE-knockout (ApoE^−/−^) mice fed an atherogenic diet for 12 weeks (n = 5, ***P < 0.005). (**c**) mRNA levels of inflammatory cytokines in the ATMs described in (**b**) (n = 5, *P < 0.05, **P < 0.01 and ***P < 0.005). (**d**) Relative mRNA levels of DNMT1 in cultured peritoneal macrophages treated with PBS or LPS (100 ng/ml) for 24 hours (n = 5, **P < 0.01). (**e**) Immunoblotting assay of DNMT1 in cultured peritoneal macrophages treated with PBS or LPS (100 ng/ml) for 24 hours. (**f**) Relative mRNA levels of DNMT1 in cultured peritoneal macrophages treated with BSA (5%) or saturated free fatty acid (sFFA, 200 μM) for 24 hours. (**g**) Immunoblotting assay of DNMT1 in cultured peritoneal macrophages treated with BSA or sFFA (200 μM) for 24 hours.

DNA methylation provides a molecular link between environmental factors (e.g., diet) and multiple diseases, including obesity and diabetes^24^,^25^. To investigate the correlation between DNA methylation and atherosclerosis (AS), we evaluated the expression levels of DNA methyltransferases in macrophages from mouse models of obesity and AS. The results showed that the mRNA levels of DNMT1, DNMT3a and DNMT3b were notably increased in the adipose tissue-derived macrophages (ATMs) from the mice fed a HFD, compared to those of mice fed a normal diet (Fig. 1a). Compared with the wild-type mice, the ApoE-knockout (ApoE^−/−^) mice, which are a well-documented AS model, displayed a notable increase in DNMT1 mRNA levels in ATMs (Fig. 1b) and peritoneal macrophages (see Supplementary Fig. S1); the mRNA levels of DNMT3a and DNMT3b were not significantly altered. We also found that the ApoE^−/−^ mouse-derived ATMs expressed more proinflammatory cytokines (e.g., TNFα, IL-1β and IL-6) and fewer anti-inflammatory cytokines (e.g., IL-10 and Arg1) than those from the wild-type mice (Fig. 1c). In addition, we demonstrated that the mRNA and protein levels of DNMT1 were dramatically induced by the obesity-related factor LPS (Fig. 1d,e) or by saturated free fatty acid (Fig. 1f,g). These findings indicated that DNMT1 was associated with inflammatory cytokine production in AS-related macrophages.

### Macrophage DNMT1 aggravates atherosclerosis development in the Tg^DNMT1^ mouse model

**Figure 2:**
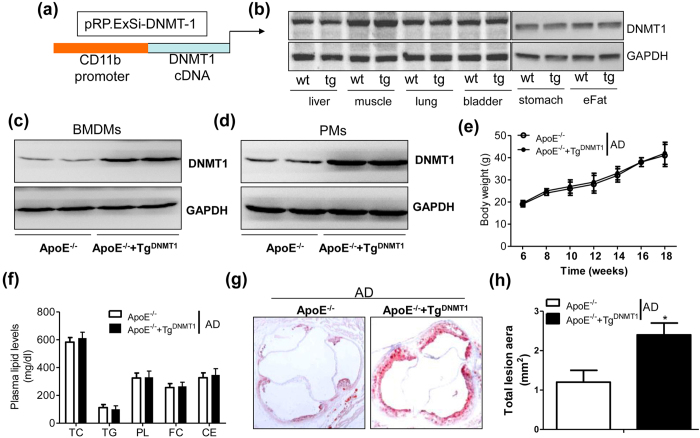
Macrophage DNMT1 aggravates atherosclerosis development in an ApoE^−/−^ mouse model. (**a**) A structural diagram of the plasmid used for the macrophage-specific transgene of mouse DNMT1. The expression of DNMT1 was specifically driven by the human CD11b promoter. (**b**) Immunoblotting assay of DNMT1 expression in multiple tissues from wild-type (wt) or Tg^DNMT1^ (tg) mice. Representative results are displayed. eFat, epididymal fat tissues. (**c**) Immunoblotting assay of DNMT1 in the bone marrow-derived macrophages (BMDM) isolated from the ApoE^−/−^ or macrophage DNMT1 transgenic (Tg^DNMT1^) ApoE^−/−^ mice. (**d**) Immunoblotting assay of DNMT1 in peritoneal macrophages (PMs) isolated from the ApoE^−/−^ or macrophage DNMT1 transgenic (Tg^DNMT1^) ApoE^−/−^ mice. (**e**) Macrophage DNMT1 transgene does not affect body weight gain. The 6-week-old ApoE^−/−^ or ApoE^−/−^ + Tg^DNMT1^ mice were fed a standard atherogenic diet (AD), and the body weight was recorded every two weeks (n = 10). (**f**) The plasma lipid levels of the ApoE^−/−^ or ApoE^−/−^ + Tg^DNMT1^ mice, which were fed a standard AD for 12 weeks (n = 10). TC, total cholesterol; TG, triglyceride; PL, phospholipid; FC, free cholesterol; CE, cholesterol ester. (**g**) Representative cross-sectional image of the aortic sinus stained with Oil Red O. (**h**) Quantification of aortic lesion area (n = 5, *P < 0.05).

To explore the role of macrophage DNMT1 in AS development, we created a mouse model that expressed a specific transgene of DNMT1 in macrophages (Tg^DNMT1^) (Fig. 2a), and we crossed those mice with ApoE^−/−^ mice. There was no significant alteration in DNMT1 expression in the organs or tissues (e.g., liver, muscle, lungs, bladder, stomach and epididymal fat) between the wild-type and Tg^DNMT1^ mice (Fig. 2b). As expected, the protein levels of DNMT1 in the bone marrow-derived macrophages and peritoneal macrophages isolated from the ApoE^−/−^-Tg^DNMT1^ mice were largely increased, compared to those from ApoE^−/−^ mice (Fig. 2c,d). These results verified that the Tg^DNMT1^ mouse model had been successfully created. After administration of a standard atherogenic diet (AD) for 12 weeks, the macrophage DNMT1 transgene had no significant effect on bodyweight gain (Fig. 2e) or plasma lipid profiles (e.g., free and total cholesterol, triglycerides, phospholipid and cholesterol esters) (Fig. 2f). However, the ApoE^−/−^-Tg^DNMT1^ mice had a significant increase in the aortic lesion area compared with that of the ApoE^−/−^ mice (Fig. 2g,h). Simultaneously, the CD68 mRNA levels of arterial plaque were notably increased in the ApoE^−/−^-Tg^DNMT1^ mice compared with those of the ApoE^−/−^ mice (see Supplementary Fig. S2a). The proportion of M1 macrophages (F4/80 + CD11c + cells) in the arterial plaque was also significantly elevated in the DNMT-transgenic group (see Supplementary Fig. S2b).

### Macrophage DNMT1 aggravates proinflammatory cytokine production

**Figure 3:**
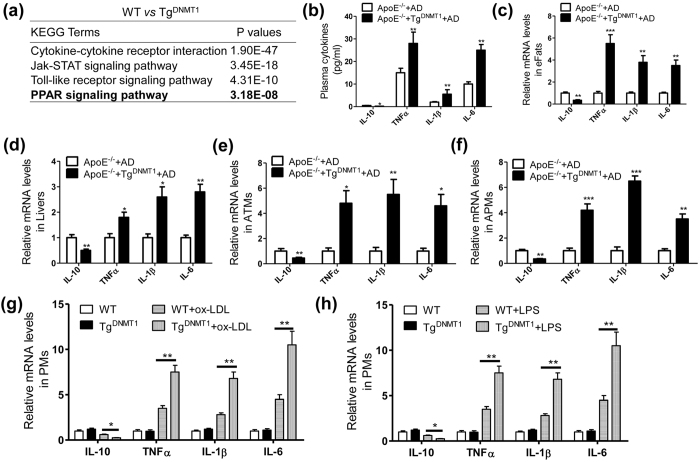
Macrophage DNMT1 aggravates proinflammatory cytokine production. (**a**) DNMT1 transgene significantly affects the inflammatory pathway and PPAR signaling pathway. Wild-type and DNMT1 transgenic peritoneal macrophages were isolated and treated with LPS (100 ng/ml) for 24 h, followed by a microarray gene assay. Then, the microarray data were subjected to KEGG analysis. The notably altered inflammatory pathways and PPAR signaling pathway, with their corresponding P values, are displayed as indicated. (**b**) Plasma cytokine levels of the ApoE^−/−^ or macrophage DNMT1 transgenic (Tg^DNMT1^) ApoE^−/−^ mice, which were fed an AD for 12 weeks (n = 6, *P < 0.5, **P < 0.01). (**c**) mRNA levels of the inflammatory cytokines in the epididymal fat tissues (eFats) isolated from the mice described in (**b**) (n = 6, **P < 0.01, ***P < 0.005). (**d**) mRNA levels of the inflammatory cytokines in the livers of the mice described in (**b**) (n = 6, *P < 0.05 and **P < 0.01). (**e**) mRNA levels of the inflammatory cytokines in the adipose tissue-derived macrophages (ATMs) from the mice described in (**b**) (n = 6, *P < 0.05 and **P < 0.01). (**f**) mRNA levels of the inflammatory cytokines in the arterial plaque-derived macrophages (APMs) from the mice described in (**b**) (n = 6, **P < 0.01 and ***P < 0.005). (**g**) mRNA levels of the inflammatory cytokines in the ox-LDL (50 nM)-stimulated peritoneal macrophages (PMs) isolated from the WT or Tg^DNMT1^ mice (n = 6, *P < 0.05 and **P < 0.01). (**h**) mRNA levels of the inflammatory cytokines in the LPS (100 ng/ml)-stimulated peritoneal macrophages (PMs) isolated from the WT or Tg^DNMT1^ mice (n = 6, *P < 0.05 and **P < 0.01).

To investigate the mechanism linking macrophage DNMT1 to the aggravation of AS, we observed the regulatory role of DNMT1 in macrophage cytokine production. Using a gene microarray analysis, we demonstrated that the DNMT1 transgene had a profound influence on the inflammatory pathways and the PPAR signaling pathway in the macrophages in response to LPS treatment (Fig. 3a). As shown in Fig. 3b–d, Tg^DNMT1^ notably increased the proinflammatory cytokines (e.g., TNFα, IL-1β and IL-6) and decreased IL-10 levels in the plasma, epididymal fat tissues and livers of ApoE^−/−^ mice that were fed an AD for 12 weeks. Furthermore, we isolated macrophages from the epididymal fat tissue and the arterial plaques of the aforementioned mice. As expected, the transgene of DNMT1 also significantly induced proinflammatory cytokine production and had the opposite effect on IL-10 expression in the macrophages from the adipose tissues (Fig. 3e) and arterial plaques (Fig. 3f). To confirm further the proinflammatory role of DNMT1 in macrophages, we isolated the PMs from wild-type or Tg^DNMT1^ mice and challenged them with the atherogenic metabolite ox-LDL^6^ or the obesity-associated factor, LPS. We found that there were no significant differences in the mRNA levels of inflammatory cytokines in the untreated PMs between the wild-type and Tg^DNMT1^ mice (Fig. 3g,h). However, in response to ox-LDL or LPS stimulation, the Tg^DNMT1^-PMs expressed notably increased levels of proinflammatory cytokines (e.g., TNFα, IL-1β and IL-6) and reduced levels of IL-10 (Fig. 3g,h). These results indicated that DNMT1 potentiated proinflammatory cytokine production in macrophages.

### DNMT1-mediated methylation of the PPAR-γ promoter

**Figure 4:**
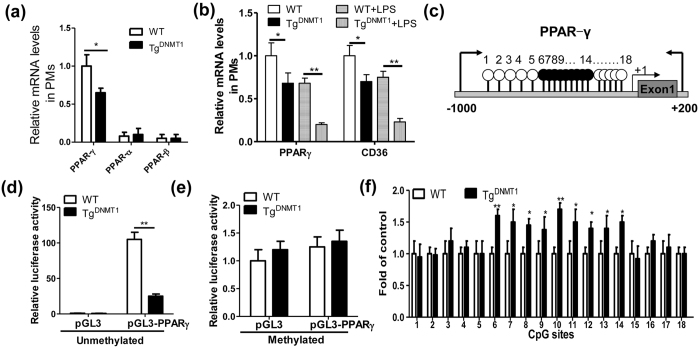
DNMT1-mediated methylation of PPAR-γ promoter. (**a**) Relative mRNA levels of PPAR-α, β and γ in the WT or DNMT1 transgenic peritoneal macrophages (n = 5, *P < 0.05). (**b**) Relative mRNA levels of PPAR-γ and CD36 in the WT or DNMT1 transgenic peritoneal macrophages treated with PBS or LPS (100 ng/ml) for 24 h (n = 5, *P < 0.05 and **P < 0.01). (**c**) Schematic illustration of the proximal PPARγ promoter region (−1000/ + 1). The transcription start site is indicated as +1. The CpG islands are indicated as upward vertical lines with open circles. (**d,e**) Promoter activity of PPAR-γ was regulated by DNMT1. Either unmethylated (**d**) or fully methylated (**e**) promoter construct pGL3 (0.4 μg/ml) or pGL3-PPARγ (0.4 μg/ml) was transfected into the PMs from the wild-type or Tg^DNMT1^ mice for 24 h. Then, the cells were harvested for luciferase assays. (n = 3, **P < 0.01). (**f**) DNA methylation levels at individual CpG sites on PPAR-γ promoter in PMs isolated from the WT or Tg^DNMT1^ mice (n = 5, *P < 0.05, **P < 0.01).

PPAR-γ is a potent anti-inflammatory factor in macrophages^11^,^12^, and it was inhibited by DNMT1 overexpression in this study, according to the gene expression microarray analysis. Using real-time PCR assays, we demonstrated that PPAR-γ was dominantly expressed in macrophages, and mRNA levels of the DNMT1 transgene were notably inhibited (Fig. 4a). Further, we confirmed that DNMT1 largely suppressed the mRNA expression of PPAR-γ and its target gene CD36 (Fig. 4b). However, the mechanism linking DNMT1 to PPAR-γ expression needs to be further elucidated.

Surprisingly, we found that the proximal promoter (−1000/ + 1) of PPAR-γ is enriched with CpG islands, according to an online prediction (http://www.urogene.org/cgi-bin/methprimer/methprimer.cgi) (Fig. 4c). This finding raises the possibility that the methylation status of PPAR-γ promoter DNA might be regulated by DNMT1. We found that the luciferase activity of the unmethylated PPAR-γ promoter was ~100-fold greater than that of the fully methylated promoter construct (Fig. 4d,e), indicating that PPAR-γ promoter activity was regulated by DNA methylation. As expected, the DNMT1 transgene notably decreased the promoter activity of PPAR-γ (Fig. 4d). Further, we also showed that the individual methylated CpG sites were mediated by DNMT1 in the PPAR-γ promoter in PMs (Fig. 4f).

### Rosiglitazone prevents macrophage DNMT1-induced proinflammatory cytokine production and AS development

**Figure 5:**
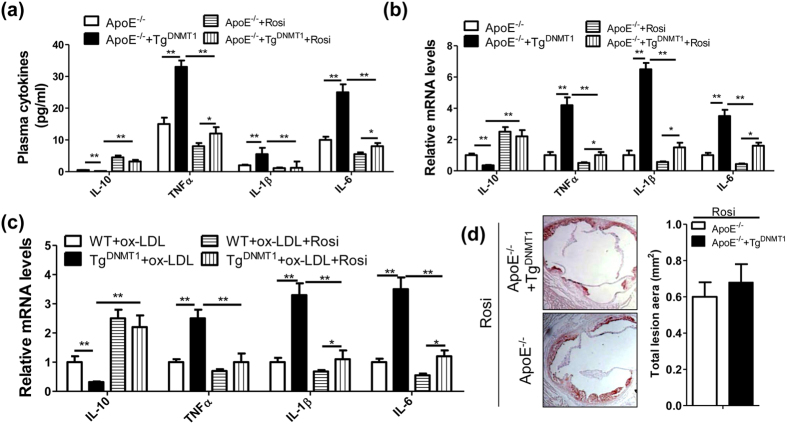
Rosiglitazone (Rosi) prevents macrophage DNMT1-potentiated proinflammatory cytokine production and AS development. (**a**) Plasma cytokine levels of the ApoE^−/−^ or macrophage DNMT1 transgenic (Tg^DNMT1^) ApoE^−/−^ mice treated with Rosi (i.p., 12 mg/100 g bw/2 days) and AD for 12 weeks (n = 6, *P < 0.05 and **P < 0.01). (**b**) mRNA levels of the inflammation-related genes in the macrophages isolated from the arterial plaques of the mice described in (a) (n = 6, *P < 0.05 and **P < 0.01). (**c**) mRNA levels of the inflammation-related genes in the ox-LDL (50 nM) and/or Rosi (50 nM)-stimulated PMs isolated from the WT or Tg^DNMT1^ mice. (n = 6, *P < 0.05 and **P < 0.01). (**d**) Representative cross-sectional image of Oil Red O-stained aortic sinuses from the indicated mice treated with Rosi (i.p., 12 mg/100 g bw/2 days) and fed an AD for 12 weeks, as well as the quantification of the aortic lesion area.

To observe whether DNMT1 regulated proinflammatory cytokine production via PPAR-γ, we used rosiglitazone, a specific PPAR-γ agonist, to rescue PPAR-γ signaling in DNMT1 transgenic macrophages. As shown in Fig. 5a, Tg^DNMT1^-induced levels of TNFα, IL-1β and IL-6 in the plasma of ApoE^−/−^ mice on an AD were largely attenuated by rosiglitazone treatment (Fig. 5a), while Tg^DNMT1^-suppressed plasma IL-10 levels were, to a great extent, rescued by rosiglitazone (Fig. 5a). Similarly, we found identical results in the macrophages isolated from the arterial plaques of the aforementioned mice with AS (Fig. 5b). Furthermore, we verified that DNMT1 regulated inflammatory cytokine production via PPAR-γ signaling in ox-LDL-treated peritoneal macrophages (Fig. 5c). *In vivo*, rosiglitazone treatment fully rescued the aggravated AS development caused by the DNMT1 transgene (Fig. 5d).

### Macrophage DNMT1 promotes proinflammatory cytokine production and AS development by suppressing PPAR-γ in macrophages

**Figure 6:**
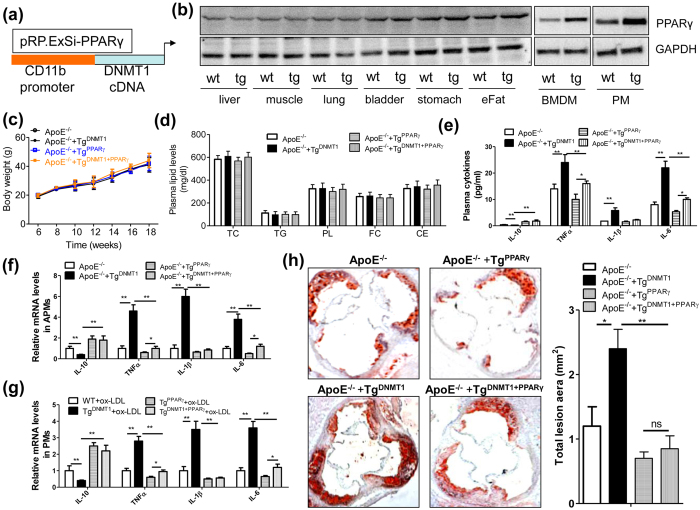
Macrophage DNMT1 promotes proinflammatory cytokine production by suppressing PPAR-γ in macrophages. (**a**) Structure diagram of the plasmid used for the macrophage-specific transgene of PPAR-γ. The expression of PPAR-γ was specifically driven by the human CD11b promoter. (**b**) Immunoblotting assay of PPAR-γ expression in multiple tissues from wild-type (wt) or Tg^PPAR-γ^ (tg) mice. Representative results are displayed. eFat, epididymal fat tissues; BMDM, bone marrow-derived macrophage; PM, peritoneal macrophage. (**c**) Macrophage transgene of PPAR-γ does not affect body weight gain. Six-week-old ApoE^−/−,^ ApoE^−/−^ + Tg^DNMT1^, ApoE^−/−^ + Tg^PPARγ^ or ApoE^−/−^ + Tg^DNMT1+PPARγ^ mice were fed an AD, and their body weights were recorded every two weeks (n = 10). (**d**) The plasma lipid levels of the above mice, which were fed an AD for 12 weeks (n = 10). TC, total cholesterol; TG, triglyceride; PL, phospholipid; FC, free cholesterol; CE, cholesterol ester. (**e**) Plasma cytokine levels in the macrophage DNMT1 and/or PPAR-γ transgenic mice fed an AD for 12 weeks (n = 6, *P < 0.05 and **P < 0.01). (**f**) mRNA levels of the cytokines in the arterial plaque-derived macrophages (APMs) from the mice described in (d) (n = 6, *P < 0.05 and **P < 0.01). (**g**) mRNA expression of the cytokines in the ox-LDL (50 nM)-stimulated PMs isolated from the WT, Tg^DNMT1^, Tg^PPARγ^ or Tg^DNMT1+PPARγ^ mice (n = 6, *P < 0.05 and **P < 0.01). (**h**) Representative cross-sectional image of Oil Red O-stained aortic sinuses from the indicated mice fed an AD and the quantification of the aortic lesion area (n = 5, *P < 0.05 and **P < 0.01).

To confirm further the role of PPAR-γ signaling in DNMT1-mediated inflammatory cytokine production, we created a macrophage-specific PPAR-γ transgenic (Tg^PPAR-γ^) mouse model (Fig. 6a,b). Similar to DNMT1, the PPAR-γ transgene in macrophages also had no significant influence on bodyweight gain (Fig. 6c) or plasma lipid profiles (e.g., free and total cholesterol, triglycerides, phospholipid and cholesterol ester) (Fig. 6d). However, Tg^DNMT1^-induced proinflammatory cytokine levels in the plasma were notably prevented by the addition of Tg^PPAR-γ^ in the ApoE^−/−^ AS model (Fig. 6e). Simultaneously, Tg^DNMT1^-reduced IL-10 levels in the plasma were rescued by Tg^PPAR-γ^ (Fig. 6e). We further verified the regulatory role of the DNMT1/PPAR-γ pathway in inflammatory cytokine production in the arterial plaque-derived macrophages (Fig. 6f) and ox-LDL-stimulated macrophages (Fig. 6f,g). To observe further the effects of the DNMT1/PPAR-γ pathway in AS development, the ApoE^−/−^-Tg^DNMT1^ mice were crossed with the ApoE^−/−^-Tg^PPAR-γ^ mice to form a double transgenic mouse model. As expected, the aggravated progression of AS caused by Tg^DNMT1^ was prevented by Tg^PPAR-γ^ (Fig. 6h).

### Elevated DNMT1 and decreased PPAR-γ levels in AS patient-associated monocytes

**Figure 7:**
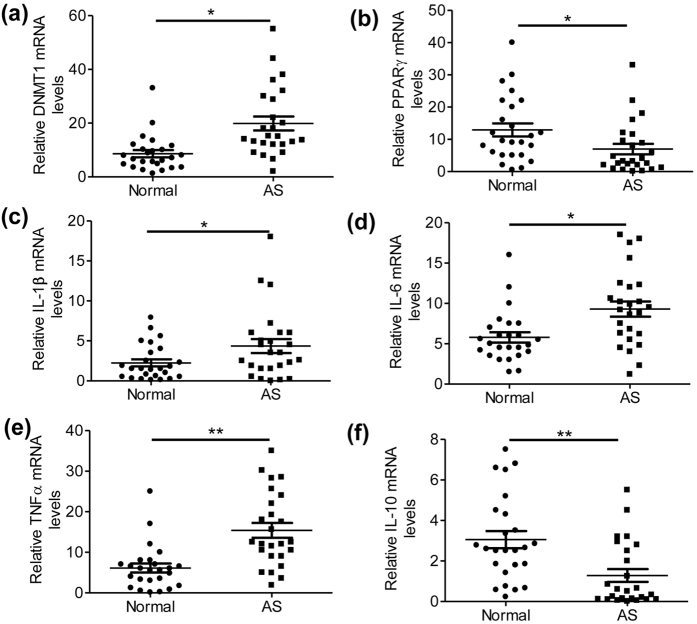
Elevated DNMT1 and decreased PPAR-γ levels in the monocytes of AS patients. (**a-f**) Relative mRNA levels of DNMT1 (**a**), PPAR-γ (**b**), IL-1β (**c**), IL-6 (**d**), TNFα (**e**) and IL-10 (**f**) in the peripheral blood monocytes isolated from the patients with AS or healthy donors (n = 25, *P < 0.05 and **P < 0.01).

To observe whether the DNMT1/PPAR-γ pathway also exists in AS patient-associated monocytes, we measured the relative expression of DNMT1, PPAR-γ and inflammatory cytokines in the peripheral blood monocytes of patients with AS and healthy donors. We demonstrated in the AS-associated monocytes that DNMT1 levels were elevated (Fig. 7a) and that PPAR-γ expression was decreased (Fig. 7b). Simultaneously, the levels of proinflammatory cytokines were increased, and the expression of anti-inflammatory cytokines was decreased in the AS group (Fig. 7c–f). In agreement, the proportion of CD14 + CD16 + cells, a type of proinflammatory monocyte^26^, increased significantly in the peripheral blood of patients with AS (see Supplementary Fig. S3a). Interestingly, the CD14 + CD16 + cells expressed higher mRNA levels of DNMT1 than did the CD14 + CD16- cells, regardless of whether the patients had AS or were normal (see Supplementary Fig. S3b). These results indicated that the DNMT1/PPAR-γ pathway might also exist in human monocytes and could regulate proinflammatory cytokine production.

## Discussion

DNA methylation has been causally linked to macrophage polarization and chronic inflammation-associated disease^17^,^27^. However, the target genes of DNA methylation and their roles in AS development remain obscure. In the present study, we identified that DNMT1 was ectopically expressed in AS-associated macrophages. Macrophage DNMT1 aggravated the development of AS by suppressing PPAR-γ-mediated anti-inflammatory effects. These findings represent potential therapeutic targets for AS.

We found that the expression of DNA methyltransferases (DNMT3a and DNMT3b) in adipose tissue-derived macrophages was induced by HFD treatment or obesity-associated factors. This finding was similar to those of a previous study^17^. In particular, we found that the induction of DNMT1, the maintenance enzyme of DNA methylation^22^,^23^, was more striking in the macrophages than the stimulation of DNMT3a and DNMT3b by HFD treatment. More interestingly, DNMT1, but not DNMT3a or DNMT3b, was induced in the ApoE^−/−^ AS model. A previous study indicated that the proinflammatory factors (e.g., saturated free fatty acid) or the anti-inflammatory cytokines (e.g., IL-4) could stimulate or suppress the expression of DNMT3b (not DNMT3a), respectively^17^. Other studies^17^,^28^ along with our findings indicated that the expression of DNA methylation-associated enzymes was individually regulated by different factors. The detailed molecular mechanisms linking HFD or ApoE to the induction of DNMT1, DNMT3a and DNMT3b remain obscure and must be very complex.

The targets of DNMT1 are universal^18^,^23^. Using microarray and KEGG analysis, we identified that PPAR signaling was notably affected by DNMT1. By further confirmation, we found that PPAR-γ, but not PPAR-α or β, was dominantly expressed and regulated by DNMT1 in macrophages. Luckily, we found that PPAR-γ, downstream of DNMT1, could effectively regulate inflammatory cytokine production in macrophages and the development of AS in the mouse model. The specific agonist of PPAR-γ also exerted a protective role in AS progression. Actually, a series of inflammatory cytokines (e.g., TNFα, IL-1β, IL-6 and IL-10) were modulated by PPAR-γ^11^,^12^. However, the cytokine that was the most important in regulating macrophage activity and AS development downstream of the DNMT1/PPAR-γ pathway remains obscure. The specific intervention of these cytokines with antibodies or knockout mouse models must be established to address this question in the future.

In summary, our data revealed the ectopic expression of DNMT1 in AS-associated macrophages. We demonstrated for the first time that DNMT1 promoted the proinflammatory activation of AS-associated macrophages and the progression of AS in mouse models. We also identified that PPAR-γ was a target of DNMT1-regulated DNA methylation and was involved in DNMT1-mediated chronic inflammation and AS development. These findings shed more light on strategies for the prevention of AS and therapies to use for AS treatment.

## Methods

### Collection and isolation of human peripheral blood monocytes

All of the experiments involving human participants were approved by the ethics committee of the Third Military Medical University and were performed in accordance with the relevant guidelines. Informed consent was obtained from all of the subjects. Human blood samples were obtained from the AS patients (age = 53 ± 7.5 years old, n = 25) and from healthy volunteers (Age = 49 ± 5.2 years, n = 25). All of the included AS patients were diagnosed at Xinqiao Hospital of Third Military Medical University. Blood samples were collected from the men before any drug treatment. Patients with AS combined with other diseases were excluded. All the steps of the blood monocyte preparation were performed at room temperature. The contents of the blood collection tubes from the same donor were pooled and mixed 1:1 with pre-warmed (room temperature) 1 × PBS (w/o Ca^2+^, Mg^2+^). The blood-PBS mixture was layered onto Lymphoprep (Axis-Shield) and was subsequently centrifuged at 800 g for 30 min at room temperature (slow acceleration, no brake). The ring-shaped interphase (peripheral blood monocytes) was collected using a Pasteur pipette into a new 50 ml tube and was diluted up to 50 ml with pre-warmed 1 × PBS (w/o Ca^2+^, Mg^2+^) and was centrifuged at 300 g for 10 min at room temperature, with fast acceleration and with brakes. The total cell number and the number of living cells were determined with Trypan blue exclusion.

### Generation of transgenic mice

The *DNMT1* or *PPAR-γ* cDNA of a mouse was subcloned into a construct that contained the human CD11b promoter to drive macrophage-specific gene expression^29^,^30^. The macrophage-specific *DNMT1* (Tg^DNMT1^) or *PPAR-γ* (Tg^PPAR-γ^) transgenic construct was microinjected into C57BL/6 embryos according to standard protocols, and the founders were crossed with the wild-type C57BL/6 strain. To rescue the PPAR-γ expression in the macrophages of Tg^DNMT1^ mice, the Tg^PPAR-γ^ mice were crossed with Tg^DNMT1^ mice to obtain double transgenic mice (Tg^DNMT1+PPAR-γ^). These experiments were approved by the Institutional Animal Care and Use Committee of the Third Military Medical University and were performed in accordance with the approved guidelines.

### Mice and diet

All of the animal experiments were approved by the Institutional Animal Care and Use of Committee at the Third Military Medical University and were performed in accordance with the “Guide for the care and use of laboratory animals” published by the US National Institutes of Health (publication no. 85–23, revised 1996). The mice were maintained in a pathogen-free facility with a 12-h light, 12-h dark cycle and were provided food and water ad libitum.

ApoE-knockout (ApoE^−/−^) mice on the C57BL/6 background were purchased from the Jackson Laboratory. Wild-type C57BL/6 mice were purchased from the Animal Center of Third Military Medical University. To investigate the role of macrophage DNMT1 or PPAR-γ in the development of AS, the Tg^DNMT1^ or Tg^PPAR-γ^ mice were mated with the ApoE^−/−^ mice. Six-week-old mice were fed an atherogenic diet (high fat-high cholesterol diet: 45% energy from lard [16:0 = 23.3%, 18:0 = 15.9%, 18:1 = 34.8%, 18:2 = 18.7%] and 0.2% [w/w] cholesterol)^31^. After 12 weeks of the atherogenic diet, the mice were sacrificed for the collection of plasma, aorta, epididymal fat and other tissues.

### Quantification of atherosclerosis

According to a previous study^32^, to analyze the development of atherosclerosis of the aortic root, the mice were euthanized after 12 weeks of feeding with the atherogenic diet. The circulatory system was perfused with PBS (100 mm Hg) for 10 min before removing the heart and aorta. The heart plus the aortic root and the aortic arch were excised, and serial sections of the aortic root were cut using a Leica CM3050 cryostat. The sections were stained with hematoxylin and 0.5% Oil Red O (Sigma-Aldrich) to evaluate the atherosclerotic intimal area of the aortic sinus. The atherosclerotic lesion area and Oil Red O-positive area were quantified using Image-Pro Plus software (Media Cybernetics).

### Extraction of peritoneal macrophages (PMs)

Primary PMs were isolated as previously described^13^. Briefly, each mouse was injected (i.p.) with 2 ml of 2.9% thioglycollate (#T9032, Sigma) on Day 1 and sacrificed on Day 3. After i.p. injection of 5 ml fresh DMEM medium, the peritoneal cells were harvested in culture dishes. Two hours later, the floating cells were washed out with PBS, and the attached cells were the PMs used for further experiment.

### Isolation of macrophages from adipose tissues or arterial plaque

Isolation of stromal vascular cells from adipose tissue was performed as previously described^13^. Isolation of macrophages (F4/80^+^ cells) from stromal vascular cells was performed by magnetic immunoaffinity isolation with anti-F4/80 antibodies conjugated with magnetic beads (MACS; Miltenyi Biotec). Cells were isolated using positive selection columns (MACS) prior to preparation of whole-cell lysates for mRNA analysis by real-time PCR.

For the collection of aortic immune cells, the aortas were harvested, cleaned, and digested to release the aortic cells, as previously described^33^. Then, the F4/80^+^ cells were selectively collected, as described above.

### Preparation of Ox-LDL

Native LDL was purchased from Sigma. Native LDL (200 mg protein/ml) was oxidized by exposure to CuSO_4_ (5 mmol/L free Cu^2+^) in phosphate-buffered saline (PBS) at 37 °C for 24 h. Control incubations were treated with 200 mmol/L EDTA. The freshly prepared ox-LDL was dialyzed at 37 °C for 48 h against 500 volumes of PBS to remove Cu^2+^, and it was sterilized by passage through a 0.45 mm filter. Oxidation of LDL was confirmed by the measurement of thiobarbituric acid-reactive substances (TBARS) with malonaldehyde bis (dimethyl acetal)^34^ as the standard. The TBARS content of ox-LDL was 6.03 ± 0.15, compared to 0.31 ± 0.12 nmol/100 mg protein in the native LDL preparation (P < 0.01). The protein content was determined using a bicinchoninic acid (BCA) protein assay kit (Pierce, Rockford, USA) with bovine serum albumin (BSA) as the standard. The ox-LDL was kept in 50 mmol/L Tris–HCl, 0.15 mol/L NaCl, and 2 mmol/L EDTA at a pH of 7.4, and it was used within 10 days of preparation.

### Bisulfite conversion and pyrosequencing

Genomic DNA was prepared by phenol/chloroform extraction. Bisulfite conversion was performed using an EpiTech Bisulfite Kit (Qiagen, Valencia, CA, USA). The primers that were used to amplify the PPAR-γ promoter covering CpG sites were commercially designed by CapitalBio Corporation (Beijing, China). Bisulfite-converted DNA (1 μg) was amplified by PCR, and pyrosequencing was performed by CapitalBio (Beijing, China).

### PPAR-γ promoter cloning and reporter gene assay

The detailed method for the DNA cloning experiments was described in a previous report^35^. The 1.0 kb DNA fragment harboring the mouse PPAR-γ proximal promoter was amplified from mouse genomic DNA using PCR. The fragment was then subcloned into a pGL3-Basic vector. The construct was confirmed by DNA sequencing. To obtain the unmethylated promoter, the reporter constructs were transformed into the *dam-/dcm- E. coli* strain (New England Biolabs, Ipswich, MA, USA). To obtain the fully methylated reporter, constructs were incubated with 3 U/μg of SssI methylase (New England Biolabs) in the presence of 160 μM S-adenosylmethionine at 37 °C for 3 hours^36^. The transfection was performed following the protocol of Lipofectamine-2000 (#12566014, Invitrogen). The luciferase activities of the cell lysates were evaluated according to the manufacturer’s instruction (#E1910, Promega), and the total protein concentration in each assay was measured as an internal control.

### Realtime PCR

Total RNAs were isolated using a peqGold Total RNA Kit including DNase digestion (Peqlab, Erlangen, Germany). RNAs were transcribed into cDNAs using Omniscript (Qiagen, Hilden, Germany). mRNA levels were determined by quantitative real-time PCR (qPCR). GAPDH was used as an internal control and gene expression levels were calculated based on the delta-Ct method. The mRNA levels for each gene represented the amount relative to the amount in the control group, which was arbitrarily standardized to one. Primer sequences used for qPCR are available upon request.

### Western blot

Tissue and cell proteins were extracted with RIPA Lysis Buffer (#P0013, Beyotime, China), and quantified with BCA kit (#P0009, Beyotime, China). The protein samples were separated by electrophoresis in 10% SDS polyacrylamide gel and transferred to a PVDF membrane (Millipore). After being blocked with 5% milk in Tris-buffered saline containing 0.1% Tween-20 (TBST), the membrane was incubated with primary antibodies for DNMT1 (#5032, Cell Signaling Technology). The antigen–antibody complex was detected using Western Lightning Plus-ECL (PerkinElmer) with GAPDH (#5174, Cell Signaling Technology) as the internal control protein. The signals were captured with a Bio-Rad ChemiDoc MP System (170-8280).

### Enzyme-linked immunosorbent assay (ELISA)

Plasma cytokines were measured with TNF-α (#MTA00B), IL-1β (#MLB00C), IL-6 (#M6000B) and IL-10 (M1000B) ELISA Kits from R&D system according to manufacturer’s protocols.

### Measurement of plasma lipids

Manufacturer’s protocols were followed to measure the levels of triglycerides (Triglyceride Reagent, Sigma #T2449; Free Glycerol Reagent, Sigma #F6428), total cholesterol (Cholesterol Liquid Reagents, Pointe Scientific Inc. #C7510-120), free cholesterol (WaKo #435-35801) and phospholipids (Phospholipid C Reagent, Wako #433-36201) in the plasma samples.

### Statistical analysis

All data are expressed as the mean ± SEM and were analyzed by either the one-way ANOVA or the unpaired Student’s two-tailed test. For each parameter of all of the data, results were significant at *P < 0.05, **P < 0.01 and ***P < 0.005.

## Additional Information

**How to cite this article**: Yu, J. *et al.* DNMT1-PPARγ pathway in macrophages regulates chronic inflammation and atherosclerosis development in mice. *Sci. Rep.* **6**, 30053; doi: 10.1038/srep30053 (2016).

## Supplementary Material

Supplementary Information
