# Supplementary material for: DNMT1-PPARγ pathway in macrophages regulates chronic inflammation and atherosclerosis development in mice
Source: Sci Rep. 2016 Aug 17;6:30053. doi: 10.1038/srep30053 (PMC4987643; doi:10.1038/srep30053)
Supplement: Supplementary Information [file srep30053-s1.pdf]

DNMT1-PPARγ pathway in macrophages regulates chronic inflammation and atherosclerosis development in mice

Jie Yu[1], Youzhu Qiu[1], Jie Yang[1], Shizhu Bian[1], Guozhu Chen[1], Mengyang Deng[1], Huali Kang[1] and Lan Huang[1]*

[1]Institute of Cardiovascular Diseases of the PLA, Xinqiao Hospital, Third Military Medical University, Chongqing, China, 400037

Supplementary Figures

Supplementary Fig. 1

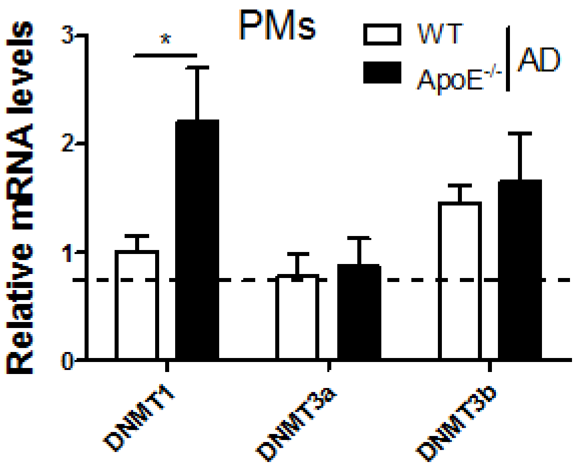

Fig. 1 Increased DNMT1 mRNA levels in the peritoneal macrophages of ApoE$^{-/-}$ mice

mRNA levels of DNMT1, DNMT3a and DNMT3b in the peritoneal macrophages (PMs) of male wild-type (WT) or ApoE-knockout (ApoE$^{-/-}$) mice fed an atherogenic diet (AD) for 12 weeks (n=5, *P<0.05).

Supplementary Fig. 2

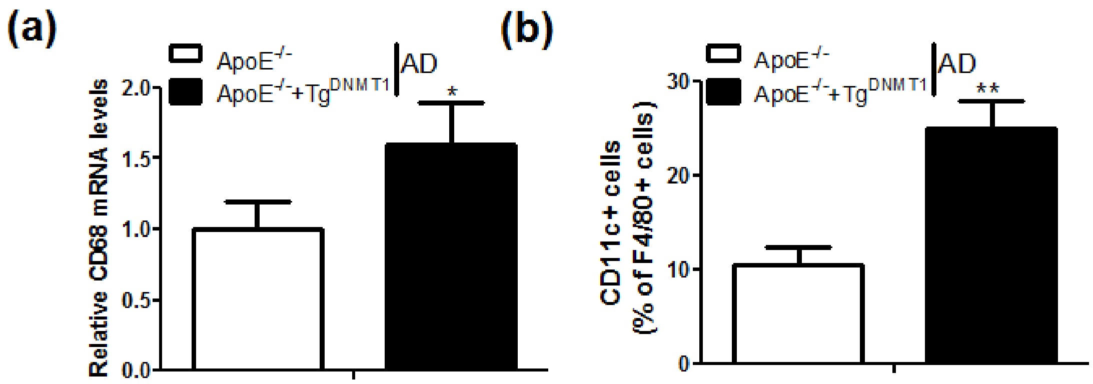

Fig. 2 Elevated ratios of M1 macrophages in the arterial plaque of Tg^{DNMT1} ApoE$^{-/-}$ mice

(a) Relative CD68 mRNA levels in the arterial plaques from the ApoE$^{-/-}$ or macrophage DNMT1 transgenic (Tg^{DNMT1}) ApoE$^{-/-}$ mice, which were fed an AD for 12 weeks (n=5, *P<0.05).

(b) Percentage of CD11c+ cells in F4/80+ cells in the arterial plaque from the mice described in (a) (n=5, **P<0.01).

Supplementary Fig. 3

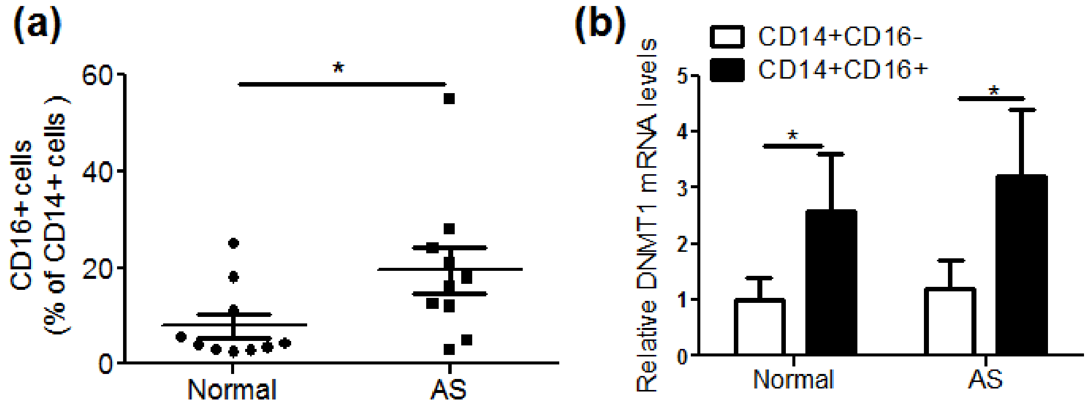

Fig. 3 Elevated ratios and DNMT1 expression of CD14+CD16+ monocytes in the blood of patients with AS

(a) Percentage of CD16+ cells in CD14+ cells in the peripheral blood monocytes isolated from the patients with AS or healthy donors (n=10, *P<0.05).

(b) Relative DNMT1 mRNA levels in the CD14+CD16- cells or CD14+CD16+ cells from peripheral blood cells of patients with AS or healthy donors (n=10, *P<0.05).
